# Effect of short-acting beta blocker on the cardiac recovery after cardiopulmonary bypass

**DOI:** 10.1186/1749-8090-6-99

**Published:** 2011-08-19

**Authors:** Jie Sun, Zhengnian Ding, Yanning Qian

**Affiliations:** 1 Dept. of Anesthesiology, the first affiliated hospital with Nanjing Medical University/Jiangsu province hospital, Nanjing 210029, P.R. China

**Keywords:** Esmolol, cardiopulmonary bypass, Mitral valve replacement, heart rate, arrhythmia

## Abstract

The objective of this study was to investigate the effect of beta blocker on cardiac recovery and rhythm during cardiac surgeries. Sixty surgical rheumatic heart disease patients were received esmolol 1 mg/kg or the same volume of saline prior to removal of the aortic clamp. The incidence of cardiac automatic re-beat, ventricular fibrillation after reperfusion, the heart rate after steady re-beat, vasoactive drug use during weaning from bypass, the posterior parallel time and total bypass time were decreased by esmolol treatment. In conclusion: Esmolol has a positive effect on the cardiac recovery in cardiopulmonary bypass surgeries.

## Background

It has been well documented that early administration of beta-adrenergic antagonist during CPB or within 10 minutes after releasing of aortic clamp contributes to left ventricular function [1,2]. Also, cardioplegia contained esmolol, an ultra-short acting (9-minute half-life) cardioselective beta blocker [3], has cardioprotection in animal model and clinical patients [4-7]. However few studies have investigated beta-adrenergic antagonist on the details of the cardiac recovery and rhythm during CPB. In this study, we therefore investigated the effect of esmolol on the cardiac recovery in rheumatic heart surgeries.

## Methods

The study protocol followed the Declaration of Helsinki and was approved by the Ethics and Research Committee of Nanjing Medical University (Nanjing, P.R. China). The study was performed in a prospective randomized manner after all the patients signed written informed consents.

60 rheumatic heart disease patients undergoing elective single mitral valve replacements were enrolled in our study. Patients were randomly assigned to two groups by a computer program. 30 patients received esmolol (Qilu Pharmacy, China)1 mg/kg prior to removal of the aortic clamp, while 28 received the same volume of saline. 2 patients were excluded because of cardiopericarditis. The cardiac recovery of patients was assessed on the basis of:(1) the heart auto re-beat ratio (the heart beat returns spontaneously without ventricular fibrillation or a temporary pacemaker); (2) the recovery time (time from reperfusion to steady heart beat); (3) the ratio of atrial fibrillation during weaning; (4) Ventricular fibrillation after primary re-beat (5) heart rate after steady re-beat; (6) heart rate 10 minutes after re-beat, and (7) temporary peri-operative pacemaking, (8) vasoactive drug use during weaning from CPB. We also recorded the bypass associated time.

### Statistical analysis

The data were analyzed with the software SPSS 11.0. The quantitative data were expressed as mean ± SD, and the difference was compared using one-factor analysis of variance. The qualitative data were compared with chi-square analysis. Fisher's exact test was used when the minimum expected count was less than five. P < 0.05 was considered to be significant.

## Results

**Figure 1:**
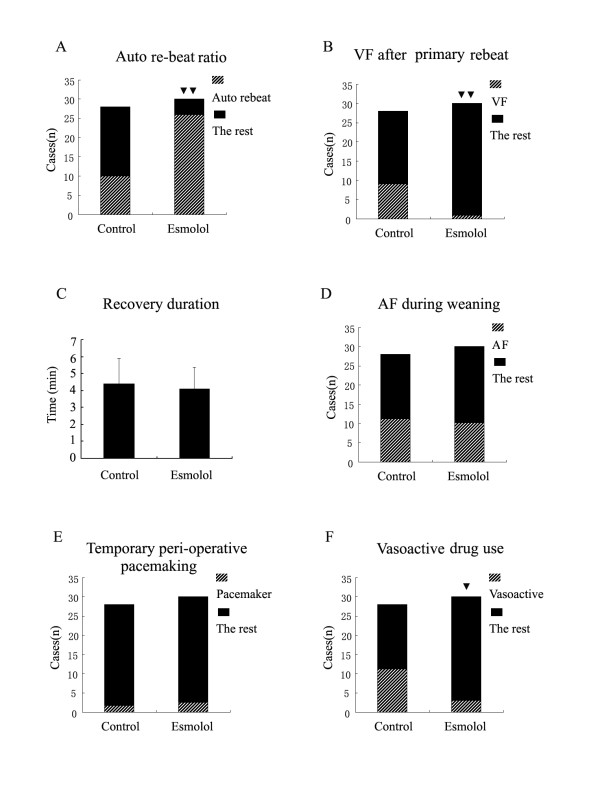
**The heart recovery data after unclamping**. A: The auto re-beat ratio in the control group and esmolol group. (two black triangles) P < 0.01 compared with control group. B: Ventricular fibrillation ratio in the control group and esmolol group. (two black triangles) P < 0.01 compared with control group. VF represents for Ventricular fibrillation. C: The recovery duration (time from reperfusion to steady heart beat) in the two groups. min represents minute. D: Atrial fibrillation during weaning from CPB. AF represents atrial fibrillation. E: Temporary peri-operative pacemaking use in the two groups. F: Vasoactive drug use in the two groups. (one black triangle) P < 0.05 compared with control group.

**Figure 2:**
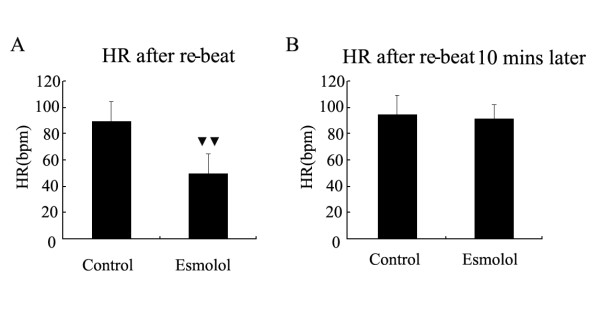
**HR after unclamping**. A: Heart rate after successful re-beat. (two black triangles) P < 0.01 compared with control group. HR represents heart rate and bpm represents beat per minute. B: Heart rate ten minutes after successful re-beat. HR represents heart rate and bpm represents beat per minute.

Following esmolol treatment, the heart underwent re-beat automatically in 26 patients, as compared to 10 patients in the control group (P < 0.001). Ventricular fibrillation after primary re-beat happened in 9 cases in control but only 1 case in esmolol group (P = 0.005). The recovery durations were 4.1 ± 1.3 min in the treatment group, and 4.4 ± 1.5 min in controls (P = 0.407). The heart rate after steady re-beat was 89.6 ± 14.9 bpm in control but 49.9 ± 14.6 in treatment group (P < 0.001). The heart rate after successful re-beat 10 minutes later was 94.8 ± 14.3 bpm in control but 91.5 ± 10.5 in treatment group (P = 0.310). Atrial fibrillation was found in 11 cases in control and 10 cases in esmolol group (P = 0.786). Two of the control group required temporary pacemaker, compared to 3 patients in the treatment group (P = 1.000). Eleven patients in control group needed vasoactive drug during weaning from bypass while only three in esmolol group (P = 0.014). (Figure 1 to Figure 2)

**Figure 3:**
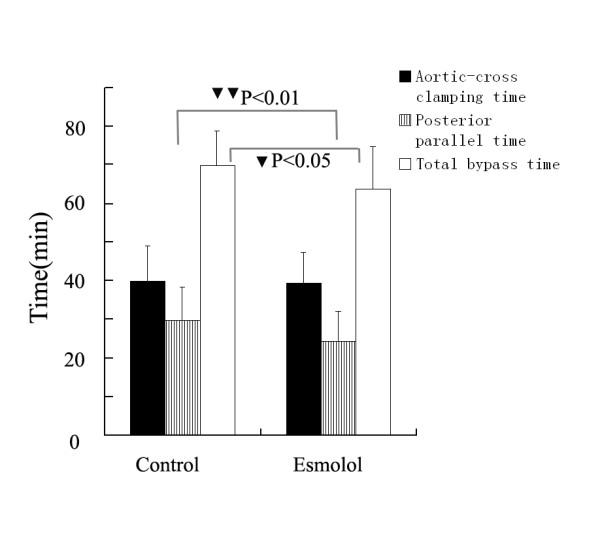
**Bypass associated time**. The bypass associated time between the groups. (one black triangle) P < 0.05 and (two black triangles) P < 0.01 compared with control group. The posterior parallel time means the time from releasing of aortic clamp to weaning from CPB.

The aortic-cross clamping time was 39.9 ± 9.1 minutes in control group and 39.4 ± 8.0 minutes in esmolol group (P = 0.827). The posterior parallel time (time from unclamping to weaning) was 29.6 ± 8.9 minutes in control group and 24.3 ± 7.8 minutes in esmolol group (P = 0.007). The bypass time was 69.9 ± 9.0 minutes in control group and 63.7 ± 10.9 minutes in esmolol group (P = 0.022). (Figure 3)

## Discussion

Our study found a positive effect of beta blocker on the prevention of ventricular arrhythmia in surgical heart disease patients. In addition, we also identified that esmolol increased the auto re-beat success rate, decreased the incidence of ventricular fibrillation after primary re-beat, and maintained better myocardial oxygen delivery and consumption balance without prolonging bypass time. On the contrary, esmolol treatment prior to removal of the aortic clamp decreased the posterior bypass time.

Esmolol treatment did not increase the need for temporary cardiac pacemaker in order to maintain the target heart rate after bypass. Because it is a very short acting beta blocker, and it seldom depresses the heart rate or contractility when administered in the early stage. In addition, esmolol improved the cardiac recovery and the heart oxygen delivery and consumption balance, which increased the myocardial energy stores and thereby benefit for the weaning process.

More and more physicians incline to use beta blocker to treat various arrhythmias including ventricular arrhythmia in non-surgical patients [8-11]. And in accordance with those studies, we also found that beta blocker had positive effect on the recovery and ventricular arrhythmias in cardiac surgery patients, which indicated beta blocker was another alternate to benefit the heart rhythm in CPB patents. Although there are some studies and meta-analysis about esmolol in cardiac surgery [12-16], most of them are about CABG patients and about peri-operative complications. Ours is more detailed on the cardiac recovery and heart rhythm during CPB intra-operatively, which is almost scarce in the present clinical researches.

In conclusion, esmolol has a positive effect on the cardiac recovery in CPB surgeries.

## List of abbreviations

CPB: cardiopulmonary bypass; CABG: coronary artery bridge grafting.

## Competing interests

The authors declare that they have no competing interests.

## Authors' contributions

First author: SJ participated in the sequence alignment and drafted the manuscript.

Second author: DZ participated in the design of the study and performed the statistical analysis.

Correspondence author: QY conceived of the study, and participated in its design and coordination.

All authors have read and approved the final manuscript.
